# Studies on the Reproductive Ability of Various Apple (*Malus × domestica* Borkh.) Cultivars Grown in the Climatic Conditions of Western Norway

**DOI:** 10.3390/plants15050701

**Published:** 2026-02-26

**Authors:** Radosav Cerović, Milica Fotirić Akšić, Milena Ðorđević, Marko Kitanović, Anđelija Obradović, Mekjell Meland

**Affiliations:** 1 Innovation Centre of Faculty of Technology and Metallurgy, University of Belgrade, Karnegijeva 4, 11120 Belgrade, Serbia; radosav.cerovic@gmail.com; 2 Department of Fruit Science, Faculty of Agriculture, University of Belgrade, Nemanjina 6, 11080 Belgrade, Serbia; fotiric@agrif.bg.ac.rs (M.F.A.); marko.kitanovic@agrif.bg.ac.rs (M.K.); 3 Department of Pomology and Fruit Breeding, Fruit Research Institute, Kralja Petra I/9, 32000 Čačak, Serbia; mdjordjevic@institut-cacak.org; 4 Institute for Science Application in Agriculture, Bulevar despota Stefana 68b, 11000 Belgrade, Serbia; aobradovic@ipn.bg.ac.rs; 5 Department of Horticulture, NIBIO Ullensvang, Norwegian Institute of Bioeconomy Research, Ullensvangvegen 1005, N-5781 Lofthus, Norway

**Keywords:** flowering time, temperature, pollen germination, pollen tube growth in vivo, fertilization success, fruit set

## Abstract

This study examines the reproductive biology of five widely cultivated apple cultivars in Norway (‘Discovery’, ‘Rubinstep’, ‘Red Aroma’, ‘Elstar’, and ‘Asfari’), when crossed with the main pollenizers (‘Summerred’, ‘Discovery’, ‘Katja’, ‘Rubinstep’, ‘Red Aroma’, ‘Fryd’, and ‘Eden’, and two crab apples ‘Professor Sprenger’ and ‘Dolgo’), as well as under self-pollination and open pollination. The experiment was conducted over two seasons (2022–2023) in Hardanger, a region in Western Norway. Flowering time and overlap, in vitro pollen germination, pollen tube growth within the styles and ovary, embryo sac viability, fertilization success, and fruit set were analyzed as key reproductive parameters. Under broadly comparable climatic conditions across both seasons, the results showed that both mother cultivar and the pollenizer strongly influenced progamic processes and fruit set. Pollen tube growth through the pistil was generally faster and more successful in 2022 for all pollination combinations, resulting in a higher fruit set. The only exception was ‘Elstar’, which exhibited a higher fruit set in 2023. If a single optimal pollenizer were to be selected for each apple cultivar in Western Norway, it would be ‘Red Aroma’ for ‘Discovery’ and ‘Rubinstep’; ‘Rubinstep’ for ‘Red Aroma’ and ‘Elstar’; and ‘Professor Sprenger’ for ‘Asfari’. Based on pollen tube growth in vivo and the fruit set, cultivars ‘Discovery’, ‘Rubinstep’, ‘Red Aroma’, ‘Elstar’, and ‘Asfari’ showed self-incompatibility.

## 1. Introduction

Apple (*Malus × domestica* Borkh.) is the main fruit crop in Norway, holding economic, commercial, social, environmental, and traditional significance [1]. Commercial apple production in Norway is concentrated in regions along the fjords in the west and southwest, as well as around lakes near the sea in the southeastern part of the country. The apple yield per hectare in Norway is significantly lower than the average yields in the world’s largest producing countries. Therefore, in 2024, the total area of apple orchards was 1640.3 ha, with a total yield of 21,662 tons and a yield of 13.21 t/ha [2]. The short and cool growing season which sometimes creates unfavorable conditions in spring and afterwards for apple flowering, can greatly reduce yields in Norwegian apple orchards.

On the other hand, these climatic conditions give the fruit a typical aroma and acidity for apples grown in the Hardanger region, which is located in Western Norway. The most popular and commercial apple cultivars are ‘Discovery’, ‘Summerred’, ‘Red Gravenstein’, ‘Red Aroma’, and ‘Rubinstep’ [3]. The market demand for new cultivars is constantly increasing, especially for those with good taste and a pleasant aroma that are easy to grow, produce large and tasty fruits, are disease-resistant, can withstand transport, and have good storage properties. Norway’s apple production is expected to increase in the near future, especially in regions once known for extremely cold climates, which are now likely to become suitable for apple growing due to more favorable heat conditions [4].

Most apple cultivars cannot self-fertilize because of a multi-allelic *S*-locus mechanism that causes gametophytic self-incompatibility. Many diploid cultivars are mainly self-incompatible, with some showing pseudo-compatibility or full compatibility, which requires insect-mediated cross-pollination for fruit and seed development [5]. Otherwise, in apple production, factors such as flower attraction [6], pollen viability [7], stigmatic receptivity [8], pollen tube growth [9], embryo sac development and viability [10,11], the effective pollination period [8], and fruit set [12], along with the occurrence of apomixis [13], are crucial for achieving high and consistent yields. Synchronization and consistency in embryo sac development are vital for the egg cell’s viability and successful fertilization. As Gillet and Gregorius [14] noted, producing viable ovules is more important for reproductive success in plants than simply producing large amounts of pollen.

To ensure consistent yields throughout an orchard, it is recommended to allocate about 10% of the area to pollenizer cultivars, which serve as pollen sources. These may include an alternative compatible apple cultivar or a specialized crabapple pollenizer cultivar [15,16]. An essential factor for successful pollination is the ratio of available pollen grains to ovules needing fertilization [17]. Pollen limitation occurs when this ratio is imbalanced, with more ovules than pollen, resulting in reduced crop yields that depend on pollination [18,19]. Low fruit and seed set can harm apple quality by causing misshapen fruits that are low in calcium and have a shorter shelf life [20]. Pollination performance significantly influences both apple quality and quantity, accounting for 65% of market production per hectare, as shown by Garratt et al. [21].

In Norway, the main limitations include the number and location of pollenizers, overlapping flowering times, and the availability of pollinators. Cool and rainy weather hampers insect activity. Climate factors, especially rising temperatures, atmospheric warming, nutrient deficits, and water stress, together with evolutionary changes that alter metabolic, physiological, and morphological traits, profoundly influence plant growth and adaptability. Plants adjust their phenological phases based on seasonal temperatures, which impacts their life cycle events. The reproductive organs of flowers are sensitive to temperature throughout development, causing a shift in cultivar distribution toward those better suited for specific temperatures. In Western Norway, extreme weather can impair apple trees’ flowering, pollination, and fruiting. A study on pears and plums showed that temperature significantly influences in vitro pollen germination, pollen tube growth, and fruit set, especially in unfavorable climates [22,23].

Apple trees are also known to suffer from water stress during the growing season, which can lead to reduced fruit set and quality, mostly due to smaller fruit at harvest and/or early fruit drop in June and August [24]. In colder apple-growing regions such as Norway, alternate bearing is a common challenge. To manage this, chemical thinning is usually applied during flowering or when fruitlets reach 10–15 mm in diameter, followed by manual thinning if necessary [25]. 

In addition to understanding the mechanisms and flow of the reproductive process, modern fruit production requires establishing the prerequisites for successful management and guidance of this process. This study evaluated flowering timing, pollen viability in vitro, fertilization dynamics, embryo sac viability, fertilization success, and fruit set in five apple cultivars (‘Discovery’, ‘Rubinstep’, ‘Red Aroma’, ‘Elstar’, and ‘Asfari’) pollinated with selected pollinizers, including self- and open pollination, under West Norwegian climatic conditions. The previous results on pears, plums, and apples demonstrated the interdependence of individual reproductive parameters within specific West Norwegian climate conditions [22,23,26,27,28,29]. It was hypothesized that the most effective pollenizer for the studied apple cultivars is characterized by a combination of key reproductive traits: the longest overlap in flowering periods chosen pollenizers with the main cultivars, high pollen viability, rapid growth of pollen tubes from the style to the ovule locules, extended embryo sac vitality, a high rate of successful fertilization, and a high number of fruit sets after artificial pollination. Identifying and recommending such pollenizers will support producers in achieving consistently high yields of high-quality fruit under the specific climatic conditions of Western Norway.

## 2. Results

### 2.1. Time of Flowering

**Figure 1:**
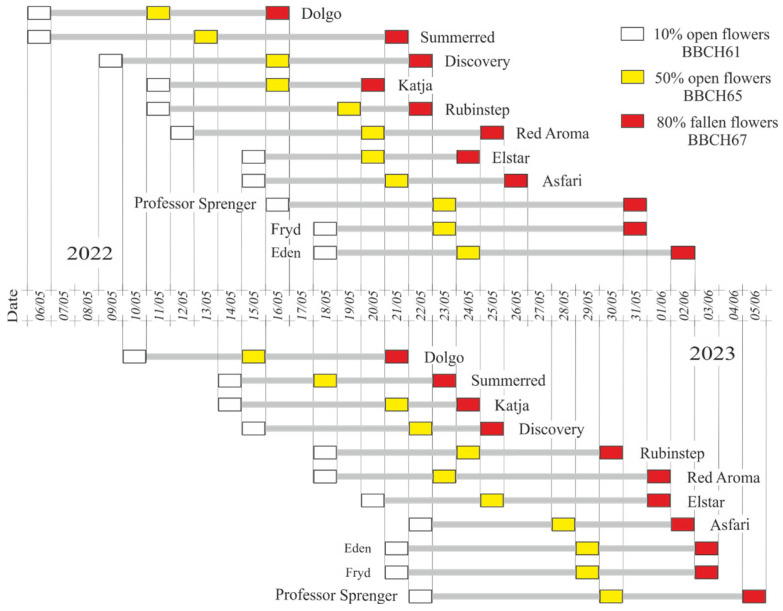
Flowering phenophases in apple cultivars in 2022 and 2023.

The flowering phenophases and their timing for each pollen recipient and pollenizer cultivar are shown in Figure 1. The start of the flowering phenophase (10% open flowers, BBCH stage 61) occurred earlier in 2022 than in 2023 for all tested cultivars. The earliest start of the BBCH stage 61 phenophase for the cultivars ‘Dolgo’ and ‘Summerred’ was recorded on 6 May in 2022, while the latest start was noted on 18 May for the cultivars ’Fryd’ and ‘Eden’. The end of effective flowering (80% fallen petals, BBCH stage 67), as defined, was recorded for the ’Eden’ cultivar on 2 June. The total duration of effective flowering for all studied cultivars, from BBCH stage 61 to BBCH stage 67 in 2022, was 28 days.

In 2023, the BBCH stage 61 flowering phenophase in cultivar ‘Dolgo’ began on 10 May, five days later than in 2022. That year, the cultivar ‘Professor Sprenger’ was the last to flower, reaching BBCH stage 67 on 5 June. The duration from the beginning of full flowering (BBCH stage 61) to the end (BBCH stage 67) was 27 days. In 2022, ‘Discovery’ had the longest flowering-phase overlap with its pollenizers, while ‘Red Aroma’ had the shortest. The cultivar ‘Discovery’ had the longest overlapping flowering period of thirteen days with ‘Summerred’, while the ‘Red Aroma‘ cultivar experienced the shortest, lasting only six days with ‘Dolgo’ pollenizer.

In 2023, the longest overlap of flowering phases, lasting thirteen days, was observed in the cultivars ‘Rubinstep’ and ‘Elstar’ with pollenizer ‘Red Aroma’, as well as in the cultivar ‘Red Aroma’ with pollenizer ‘Rubinstep’. In contrast, the shorter overlap of flowering phases in the cultivars ‘Rubinstep’ and ‘Red Aroma’ with pollenizer ‘Dolgo’ lasts only four days.

### 2.2. Air Temperature and Precipitation

**Figure 2:**
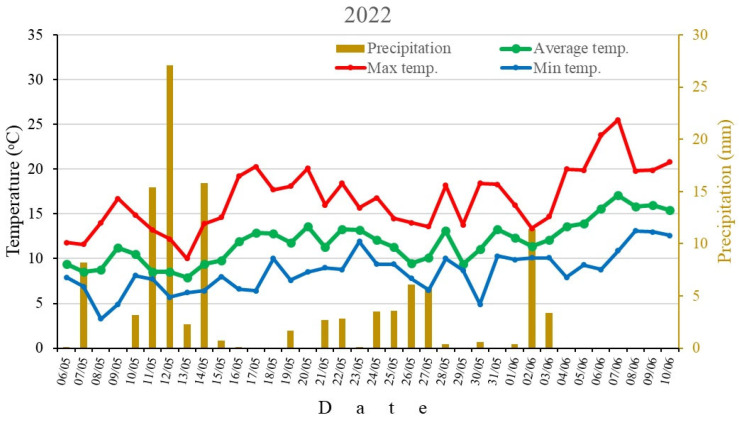

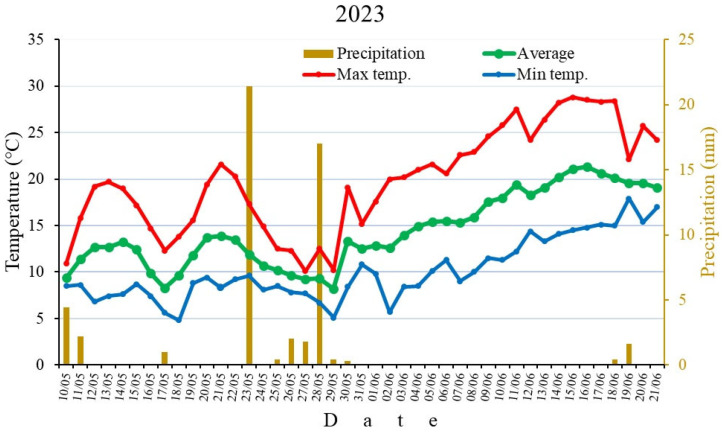
Daily temperatures and precipitation during the flowering period in 2022 and 2023.

Values of temperature and precipitation during the flowering period needed for pollen tube growth in vivo in all pollination combinations, plus period for embryological studies, for 2022 and 2023 are shown in Figure 2. 

Flowering period in 2022 lasted 36 days, from the opening of the earliest cultivar’s flowers to the petal fall of the latest cultivar. The average daily air temperature in 2022 was 11 °C. That year, the mean maximum temperature reached 23.8 °C, while the minimum was 3.3 °C. Precipitation occurred on 22 days during this period, totaling 115.5 mm. During the period of pollen tube growth in vivo in the pistil, the temperature was slightly higher, averaging 11.6 °C across all pollen recipient apple cultivars, with a total average precipitation of 26.3 mm. The period for embryological studies was characterized by an average temperature of 12.7 °C, with a higher total average precipitation of 42 mm.

The following year, the average daily air temperature during the flowering period for the examined apple cultivars was 14.4 °C. The highest temperature recorded was 28.8 °C, while the lowest was 4.8 °C. Precipitation occurred for only 12 days, totaling 52.9 mm. The average daily temperature during pollen tube growth in vivo in the mother cultivars was 11.8 °C. During this period, the total average precipitation was 34.4 mm. Additionally, during a 21-day period after full flowering (for embryological studies), the average temperature was 13.6 °C, with a higher total average precipitation of 36.2 mm.

### 2.3. Pollen Germination In Vitro

**Figure 3:**
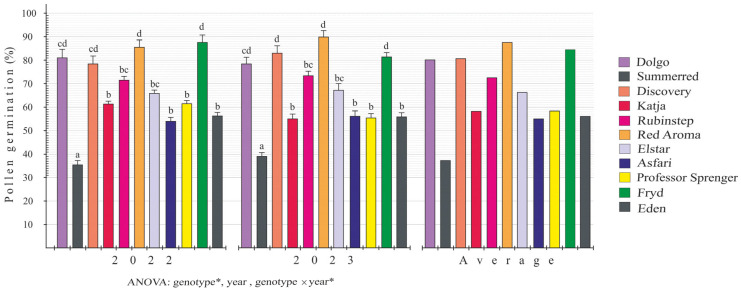
In vitro pollen germination (% ± SD) of apple cultivars in 2022 and 2023. Different letters above the bars denote a significant difference between cultivars according to the Tuckey test, *p* < 0.05; *—significant differences.

Tests for in vitro pollen germination and pollen tube growth are key indicators of pollen viability. The results of the in vitro pollen germination study are shown in Figure 3. Statistical analysis of the data revealed significant differences among cultivars and, in some cases, an interaction between cultivar and certain years. On average, across all studied cultivars, the highest pollen germination percentages were observed in ‘Red Aroma’ (87.8%), ‘Fryd’ (84.7%), and ‘Discovery’ (80.8%), while the lowest were in ‘Summerred’ (37.3%), ‘Asfari’ (55.1%), and ‘Eden’ (56.1%). Furthermore, by year, the ‘Red Aroma’ cultivar displayed the highest pollen germination rate at 89.9 ± 2.8% in 2023, while the ‘Summerred’ cultivar had the lowest at 35.5 ± 1.8% in 2022.

### 2.4. Pollen Tube Growth In Vivo

**Figure 4:**
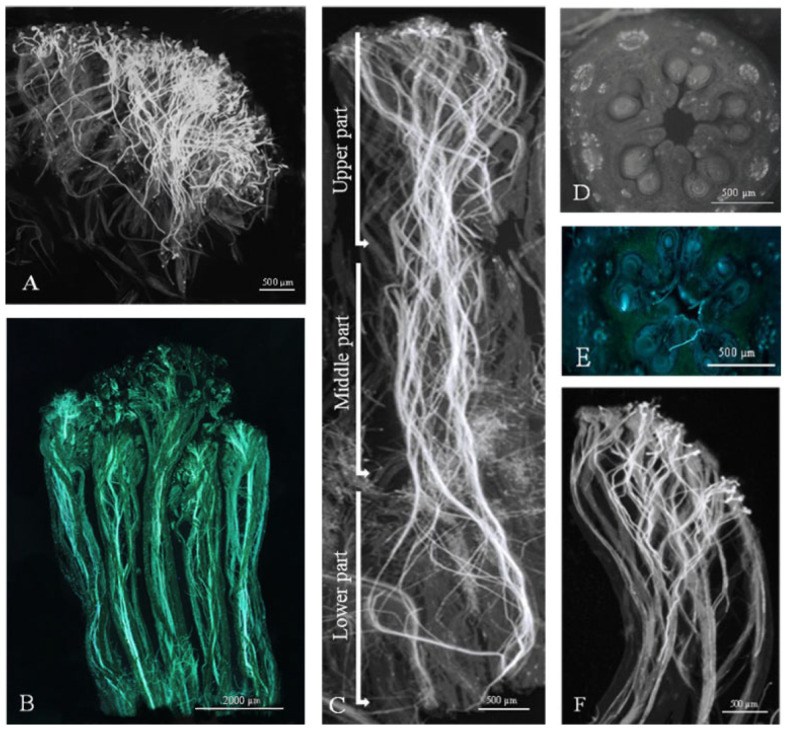
(**A**) Germination of pollen grains and the penetration of pollen tubes into the surface of the stigma. (**B**) Pollen tube growth through all five styles. ‘Red Aroma’ × ‘Dolgo’ ten days after pollination. (**C**) The growth of the pollen tube in the upper, middle, and lower part of the individual style. ‘Discovery’ × ‘Katja’ six days after pollination. (**D**) The ovary has five locules, each containing two ovules. (**E**) The growth of pollen tubes within the locules of the ovary. ‘Asfari’ × ‘Eden’ six days after pollination. (**F**) Arrested growth of incompatible pollen tubes in the upper part of the style. ‘Elstar’ six days after self-pollination.

In our studies, it was common to observe an overload of pollen grains on the stigma surface (Figure 4A). Pollen tube growth in vivo begins with pollen germination on the moist stigma, which has small to medium-sized papillae and secretory fluid filling the gaps. The adhesion of pollen to the stigma, followed by its interaction with the papillae, leads to germination, specifically the penetration of the pollen tube through the stigma. Pollen tubes then penetrate extracellularly through the ‘transmitting tissue’ of all five styles in a progressive manner, ‘bound’ in an intercellular way (Figure 4B).

This ‘transmitting tissue‘ belongs to the solid or closed type, forming a funnel-shaped surface at the top of the style and being in direct contact with the stigma papillae. Additionally, the cells of ‘transmitting tissue’ are elongated and have large intercellular spaces. During this growth process, the number of pollen tubes decreased significantly as they grew through different parts of the style—upper, middle, and lower (Figure 4C). Pollen tube growth continues through fused styles, reaching the individual obturators and ultimately entering the ovary locules. The pentacarpelar apple gynoecium consists of five ovary locules, each containing two anatropous ovules (Figure 4D). After traveling through the funiculus and placental tissue, the pollen tubes make a 180° turn inside each locule (Figure 4E). This maneuver allows the pollen tube to penetrate over the obturator to the micropyle of the ovule. In cases of self-pollination, incompatible pollen tubes stop growing in the upper part of the style (Figure 4F).

**Figure 5:**
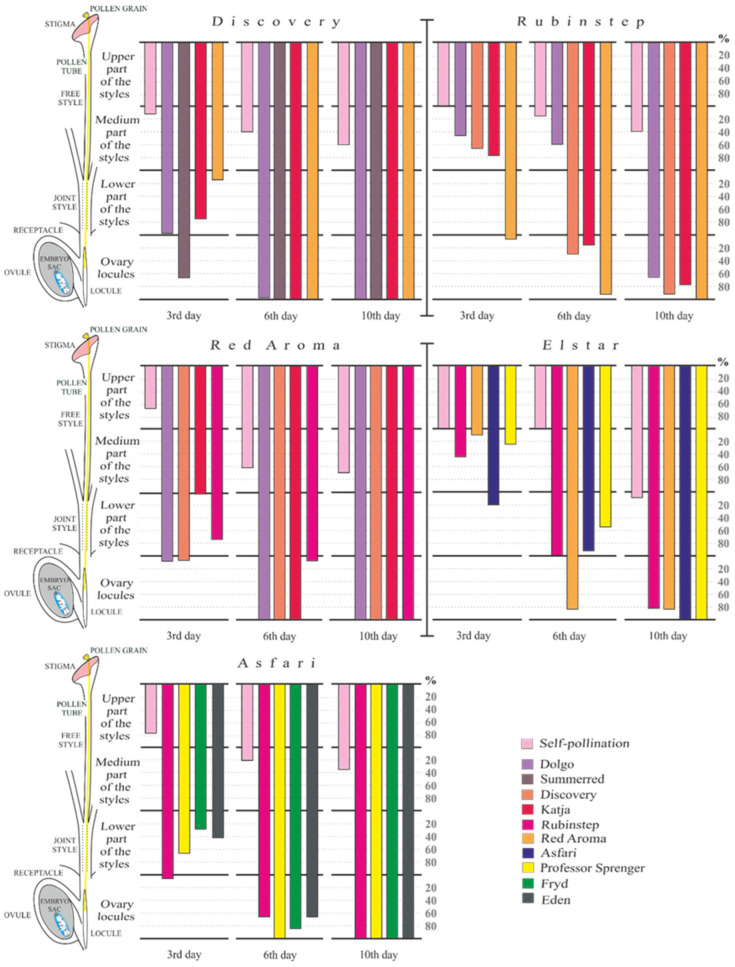
Dynamic pollen tube growth through different pistil parts of five apple cultivars under various pollination combinations in 2022.

The dynamics of pollen tube growth in vivo across all pollination combinations among five apple cultivars in 2022 are depicted in Figure 5. Cultivar ‘Discovery’ in crosses with ‘Summerred’, ‘Katja’, and ‘Red Aroma’ reached a maximum pistil percentage of 100%, with pollen tubes arriving at the ovary locules by the sixth day after pollination in 2022. The pollination combination of ‘Discovery’ × ‘Dolgo’ showed a slightly lower percentage (93.8% pistils) during the same period. 

Subsequently, ‘Red Aroma’, when pollinated with ‘Dolgo’, ‘Discovery’, and ‘Katja’, also reached the maximum pistil percentage, with pollen tubes penetrating the ovary locules six days after pollination. Both pollen recipient cultivars, ‘Discovery’ and ‘Red Aroma’, achieved 100% pistils with pollen tube penetration in the ovary locules across all pollination combinations by the tenth day after pollination. In the pollen recipient ‘Asfari’, the combination with ‘Professor Sprenger’ showed the most effective pollen tube growth. By the sixth day after full flowering, 100% of pistils had pollen tubes penetrating the ovary locules. Ten days after pollination, the other three pollination combinations—‘Asfari’ with ‘Rubinstep’, ‘Fryd’, and ‘Eden’—also showed the highest percentage of pistils with pollen tube penetration into the ovary locules. For the pollen recipient ‘Elstar’, on the tenth day after pollination, two crossing combinations (with ‘Asfari’ and ‘Professor Sprenger’) had the maximum percentage of pistils with pollen tube penetration into the ovary locules. Similarly, the pollen recipient ‘Rubinstep’ had maximum pollen tube penetration only when pollinated with ‘Red Aroma’.

**Figure 6:**
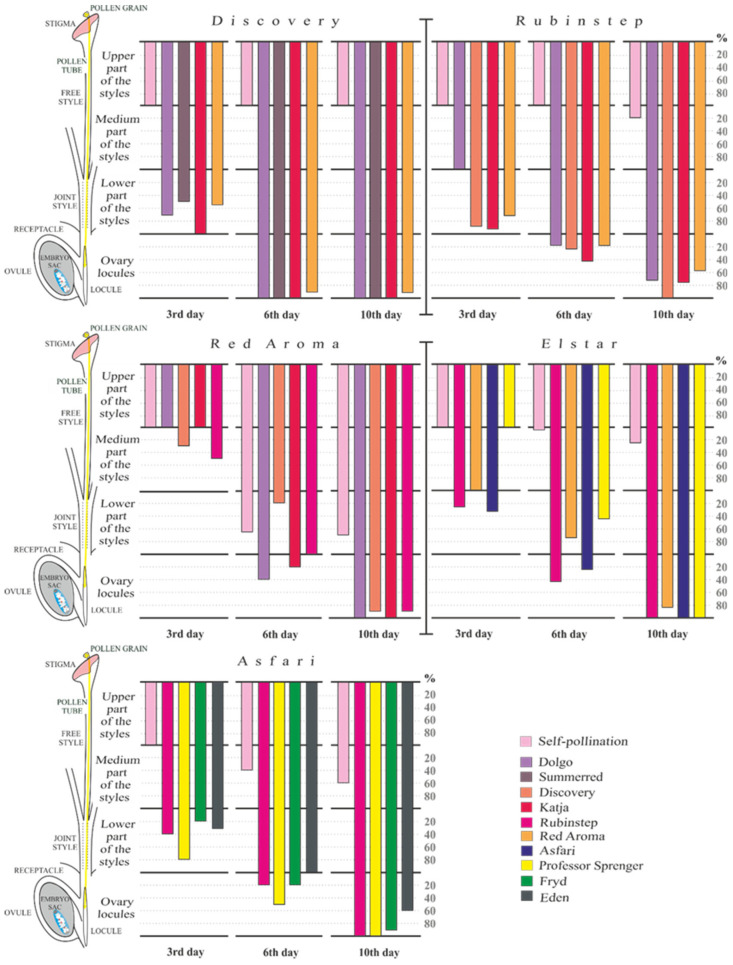
Dynamic pollen tube growth through different pistil parts of five apple cultivars under various pollination combinations in 2023.

The dynamics of pollen tube growth in 2023 across all pollination combinations are shown in Figure 6. This year, cultivar ‘Discovery’ exhibited the most effective pollen tube growth among all pollination combinations, compared with the other four pollen-recipient cultivars. On the sixth day after pollination, the highest percentage of pistils (100%) with pollen tubes penetrating the ovary locules of cultivar ‘Discovery’ was observed across three pollination combinations (‘Dolgo’, ‘Summerred’, and ‘Katja’). In the ‘Discovery’ × ‘Red Aroma’ combination, the percentage was slightly lower, reaching 92.7% of pistils with pollen tubes in ovary locules by the tenth day after pollination. All other pollen recipient cultivars reached maximum pollen tube penetration on the tenth day after pollination. The cultivar ‘Elstar’ shows the most significant pollen tube penetration when crossed with ‘Rubinstep’, ‘Asfari’, and ‘Professor Sprenger’, but lower when pollinated with ‘Red Aroma’ (81.8%). Additionally, ‘Red Aroma’ exhibited 100% of pistils with pollen tubes penetrating ovary locules in two pollination combinations (with ‘Dolgo’ and ‘Katja’) ten days after full flowering, while 90% of pistils had pollenizers ‘Discovery’ and ‘Rubinstep’. The ‘Asfari’ cultivar exhibited 100% of pistils with pollen tubes penetrating the ovary locules after the tenth day of pollination in two combinations (with ‘Rubinstep’ and ‘Professor Sprenger’). During the same period, when crossed with pollenizers ‘Fryd’ and ‘Eden’, this cultivar reached 90% and 60% of pistils with pollen tubes in the ovary locules, respectively. Mother cultivar ‘Rubinstep’ had the lowest efficiency of pollen tube growth dynamics through different parts of the pistil. In the crossing combination of ‘Rubinstep’ × ‘Discovery’, the penetration of pollen tubes into ovary locules was recorded in 100% of pistils on the tenth day after pollination. In the other three combinations, it reached 58.3%, 72.7%, and 76.9% when crossed with ‘Red Aroma’, ‘Dolgo’, and ‘Katja’, respectively.

In self-pollination combinations across all five pollen-recipient cultivars, the effectiveness of tube growth through specific parts of the pistil was low but varied among the cultivars. In the first year, the maximum penetration of pollen tubes into the lower part of the style was observed only in ‘Elstar’, occurring in 9.1% of pistils on the tenth day after full flowering. In the second year, in that region, pollen tubes were observed in the ‘Red Aroma’ cultivar in 70% of pistils. Conversely, in both years, pollen tubes mostly stopped in the medium part of the styles across all other self-pollination combinations.

### 2.5. Developing and Viability of Embryo Sac

**Figure 7:**
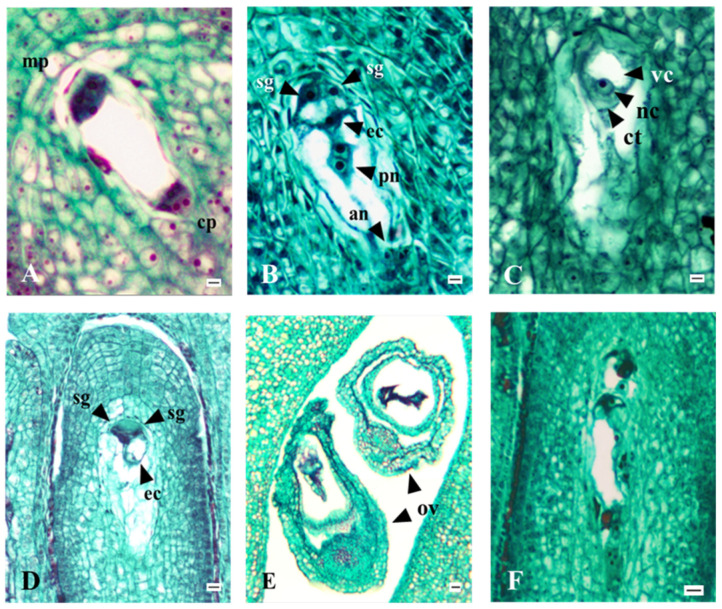
(**A**) The embryo sac after second mitotic division during process of megagametogensis—‘Discovery’; (**B**) The position of the synergid cells, egg cell, polar nuclei, and remains of the antipodes in the embryo sac—‘Rubinstep’; (**C**) The egg cell—‘Elstar’; (**D**) Degeneration of synergids—‘Red Aroma’; (**E**) Complete degeneration of ovules—‘Elstar’; (**F**) Irregular embryo sac—‘Asfari’; mp: micropylar pole; cp: chalazal pole; arrowheads (sg: synergid; ec: egg cell; pn: polar nuclei; an: rest of antipodes; vc: vacuole; nc: nucleus; ct: cytoplasm; ov: ovule); Scale bars = 10 µm.

The apple ovary contains five locules, with two ovules in each. The anatropic, bitegmic, crassinucellate ovules, all of similar size, have short funiculi. In our experiment, the process of megasporogenesis was not observed because it occurred before the onset of flowering. During the early stages of embryo sac development, through the process of megagametogenesis, eight nuclei are produced through three mitotic cycles, ultimately leading to the formation of an 8-nucleate *Polygonum* embryo sac (Figure 7A). This embryo sac contains two pear-shaped synergids with large vacuoles at the chalaza end, and nuclei positioned above them (Figure 7B). The egg cell, located between and slightly below the synergids, has a large vacuole and a nucleus within the sickle-shaped cytoplasmic zone at the chalazal end (Figure 7C). The central cell contains two polar nuclei, which are positioned near each other, while three transient antipodal cells are at the chalazal end of the embryo sac (Figure 7B).

At a later stage of full flowering, the polar nuclei fuse into a single central nucleus, while the three antipodal cells degenerate, and the 5-nucleate embryo sac is formed. Before syngamy, the embryo sac commonly has four nuclei; at this stage, the typical elongation of the embryo sac toward the chalazal end of the ovule is observed (4-nucleate embryo sac). Additionally, during the flowering stages, embryo sacs with three (Figure 7D), two, or only one nucleus were observed. These stages of the embryo sac are nonfunctional and typically comprise two polar nuclei and an egg cell, or two polar nuclei alone. Along with the appearance of these nonfunctional stages, signs of embryo sac degeneration may also be observed. The degeneration of individual elements of the embryo sac is characterized by the loss of their usual shape, which also affects the ovules themselves (Figure 7E). Occasionally, the egg sac exhibited a strong stain reaction, indicating complete degeneration. In both years of this study, irregular spatial arrangements and atypical shapes of individual embryo sac elements were observed (Figure 7F).

**Figure 8:**
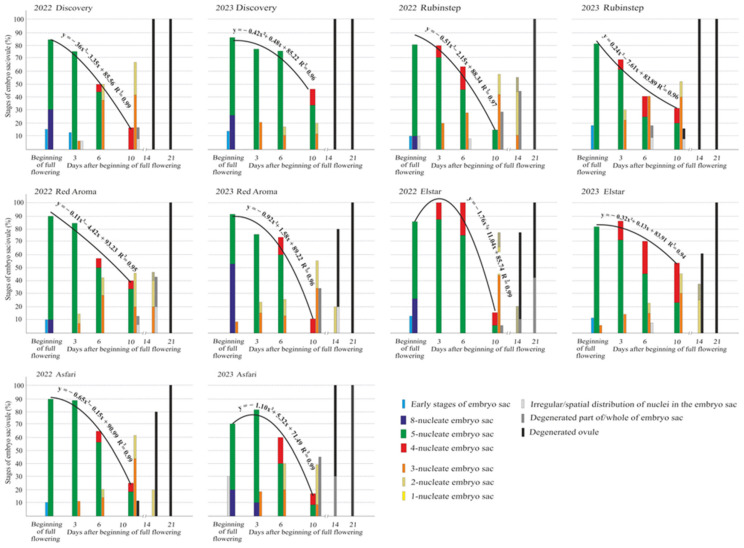
The functional stages of the embryo sac (%) and the embryo sacs/ovule (%) exhibiting degenerations in unpollinated flowers at the beginning of full flowering and on the third, sixth, tenth, fourteenth, and twenty-first day afterward. A regression analysis of the viability of the embryo sac (showing 8-, 5-, and 4-nucleate functional stages) from the beginning of full flowering to the tenth day is provided.

Figure 8 shows the viability of the embryo sac, indicated by the presence of functional stages (8-, 5-, and 4-nucleate), from the beginning of full flowering (BFF) until the twenty-first day afterwards in unpollinated flowers over two years. Early stages of embryo sac development were observed only at the BFF across all cultivars and both years, except for ‘Red Aroma’ in 2023. The highest percentage at this stage (18.2%) was seen in ‘Rubinstep’ in 2023. This study found that functional embryo sac stages (8-, 5-, and 4-nucleate) were present from BFF until 10 days in all cultivars and both years.

In unpollinated flowers, all three functional stages of the embryo sac capable of fertilization appeared cumulatively, with a parabola-like regression line showing the trend of embryo sac viability during flowering. The multiple correlation coefficients (R^2^), ranging from 0.94 to 0.99, indicated a strong link between the overall functional stages of the embryo sac and the days of flowering in both years across all studied cultivars.

The regression trend in both years indicated that an initially high percentage of functional embryo sacs at full bloom does not necessarily lead to higher percentages of functional, longer-viable embryo sacs later in full flowering. A decline in embryo sac function was observed between the sixth and tenth days after BFF in both years across all cultivars. Additionally, the regression trend for the cumulative viability of functional embryo sacs in ‘Red Aroma’ and ‘Asfari’ showed higher values in 2022 than in 2023. In that year, on the tenth day after BFF, ‘Red Aroma’ reached the highest number of functional embryo sac stages (5- and 4-nucleate embryo sacs) in 40% of the examined ovules, while ‘Elstar’ had the lowest at 16.7%. Conversely, in 2022, ‘Elstar’ had 100% functional embryo sacs on the third and sixth days after BFF. Moreover, the regression trends for this cultivar indicated a faster decline in the percentage of viable embryo sacs. In 2023, the cumulative viability regression trend for ‘Discovery’, ‘Rubinstep’, and ‘Elstar’ was higher than in 2022. On the tenth day after flowering started, ‘Elstar’ had the highest percentage of functional embryo sacs (5- and 4-nucleate) at 53.9%, while ‘Red Aroma’ had the lowest at 11.1%.

After the tenth day from BFF, there were no vital functional embryo sacs (8-, 5-, or 4-nucleate) in any mother cultivar (Figure 8). In addition to embryo sacs at functional stages, nonfunctional embryo sacs (3-, 2-, or 1-nucleate) were present in all cultivars in both years. At these stages, some parts of the egg apparatus were missing (one or two synergids, egg cells, or polar nuclei), making the egg incapable of fertilization. These embryo sacs were observed in all apple cultivars beginning on day 3 after BFF, with the highest numbers recorded on the sixth or tenth day after BFF, depending on the cultivar and year. Additionally, on the fourteenth and twenty-first days after BFF, a high percentage of degeneration of some part of the embryo sac, the entire embryo sac, or even the entire ovule was observed across all cultivars in both years. In ‘Discovery’ in both years, fourteen days after BFF, and in ‘Rubinstep’ in 2023, 100% of pistils showed complete degeneration of the ovules.

### 2.6. Fertilization Success

**Figure 9:**
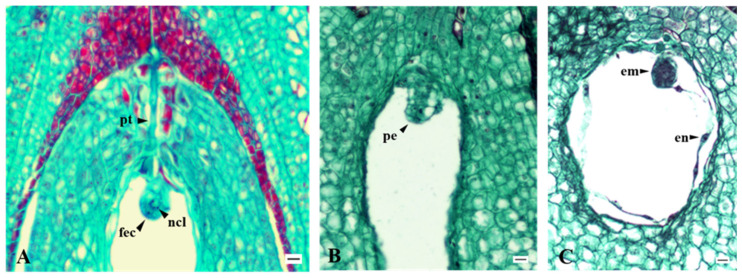
(**A**) Pollen tube that has penetrated the embryo sac and early division of a fertilized egg cell—‘Red Aroma’; (**B**) Proembryo—‘Discovery’; (**C**) Globular embryo with endosperm—‘Asfari’; arrowheads (pt: pollen tube; fec: fertilized egg cell; ncl: nucleoli; pe: pro-embryo; em: embryo; en: endosperm nuclei); Scale bars = 10 µm.

Fertilized egg cells and different early embryo structures were observed in accordance with their ontogenetic development (pro-embryo and globular embryo). It is common for one synergid to degenerate before the pollen tube reaches the embryo sac. A fertilized egg cell is larger, more colored, and has more nucleoli (Figure 9A). The formation of various early embryonic structures and the appearance of endosperm are critical stages in early embryogenesis (Figure 9B,C). Endosperm formation generally overlaps with embryo morphogenesis. The endosperm at this stage is coenocytic.

**Figure 10:**
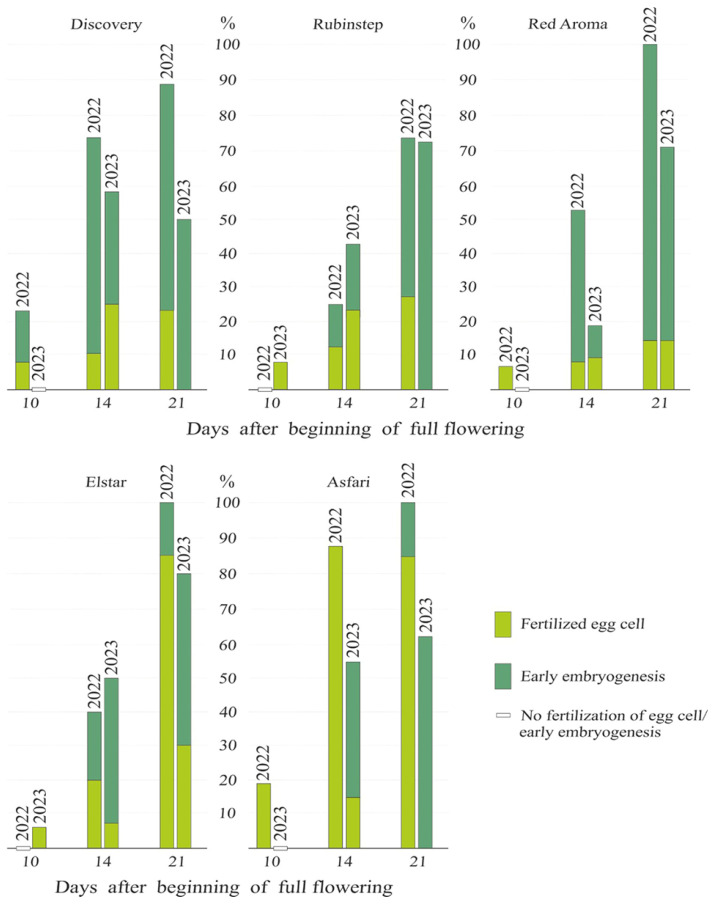
Percentage of embryo sacs with fertilized egg cell and early embryogenesis in open-pollination of five apple cultivars during 2022 and 2023. The standard deviation (SD) of the studied traits, expressed as percentages, was 2–3%.

Fertilization success, defined as the percentage of embryo sacs with a fertilized egg cell or early embryogenesis in apple cultivars, was examined throughout the entire flowering period (from BFF until twenty-one days after) under open-pollination conditions. These embryo sacs were observed after the tenth day from the BFF in both years of the study and across all cultivars (Figure 10). In 2022, ten days after the beginning of full flowering, embryo sacs with fertilized egg cells (6.7% and 18.8%) were found in the two cultivars ‘Red Aroma’ and ‘Asfari’, respectively. That year, ‘Discovery’ showed the highest fertilization success, with 15.4% of pistils containing embryo sacs with early embryogenesis. By the fourteenth day, both fertilization success parameters (fertilized egg cell and early embryogenesis) were observed across all cultivars at varying levels. When summing these two parameters, the highest percentage of pistils in 2022 was observed for ‘Red Aroma’, ‘Elstar’, and ‘Asfari’ (100%), whereas the lowest was recorded for ‘Rubinstep’ (72.8%). ‘Red Aroma’ also had the highest number of pistils with embryo sacs showing early embryogenesis (85.7%). In the following year, only ‘Rubinstep’ and ‘Elstar’ showed embryo sacs with fertilized egg cells on the tenth day after BFF (7.7% and 6.3%, respectively).

As in the previous year, both fertilization success parameters were measured for all cultivars on the fourteenth day after BFF in 2023. That year, ‘Elstar’ had the highest fertilization success rate (80%), whereas ‘Discovery’ had the lowest (50%). However, when the evaluation is restricted to the embryo sac stage with early embryogenesis, ‘Rubinstep’ has the highest proportion of pistils at this stage (72%). Otherwise, all cultivars exhibited higher fertilization success in 2022 than in 2023.

### 2.7. Fruit Set

**Figure 11:**
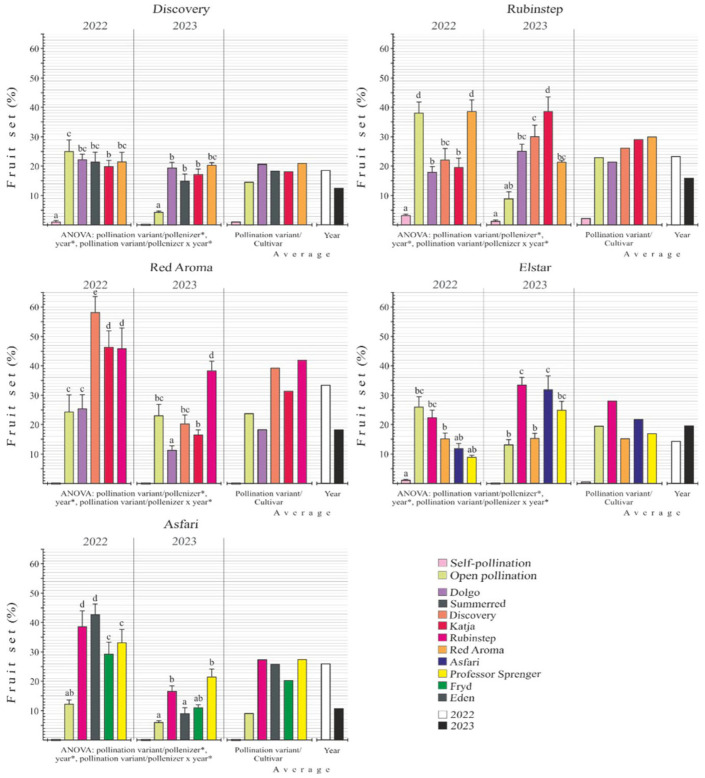
Final fruit set (% ± SD) in five apple cultivars in various pollination combinations in 2022 and 2023. Different letters above the bars denote a significant difference between cultivars according to the Tukey test, *p* < 0.05; *—significant differences.

The fruit set values for the five pollen recipients—‘Discovery’, ‘Rubinstep’, ‘Red Aroma’, ‘Elstar’, and ‘Asfari’—across all pollination combinations, including self- and open pollination for both 2022 and 2023, are illustrated in Figure 11. All examined factors—pollination combination, year of study, and their interactions—significantly affected fruit set. When averaging results from five mother cultivars for all pollination combinations, the average final fruit set in 2022 was 23.2%, compared to 16.3% in 2023. In fact, this average was higher in 2022 for four pollen recipient cultivars (’Discovery’, ‘Rubinstep’, ‘Red Aroma’, and ‘Asfari’), whereas for ‘Elstar’ it was higher in 2023.

In 2022, the pollen recipient ‘Red Aroma’ had the highest average fruit set across all pollination combinations, at 33.4%, followed by ‘Asfari’ at 26.1%, ‘Rubinstep’ at 21.3%, ‘Discovery’ at 18.6%, and ‘Elstar’ at 14.3%. Among pollination combinations with tested pollenizers, ‘Red Aroma’ × ‘Discovery’ achieved the highest fruit set that year, at 58.2 ± 5.5%. Among the other pollen recipient cultivars, the following pollination combinations showed the highest single fruit set: ‘Asfari’ × ‘Eden’ (42.9 ± 3.7%), ‘Rubinstep’ × ‘Red Aroma’ (38.7 ± 5.5%), ‘Elstar’ × ‘Rubinstep’ (22.5 ± 2.0%), and ‘Discovery’ × ‘Dolgo’ (22.3 ± 2.0%). The lowest fruit set among these combinations was ‘Elstar’ × ‘Professor Sprenger’ (9.0 ± 0.8%). For other pollen recipient cultivars, the lowest fruit set values were: ‘Rubinstep’ × ‘Dolgo’ (18 ± 2.0%), ‘Discovery’ × ‘Katja’ (19.0 ± 3.3%), ‘Red Aroma’ × ‘Dolgo’ (25.4 ± 4.8%), and ‘Asfari’ × ‘Fryd’ (29.4 ± 4.0%). Under open pollination, the highest fruit set was in ‘Rubinstep’ at 38.1 ± 6%, and the lowest in ‘Asfari’ at 12.3 ± 1.5%. In the self-pollination variant, fruit set was very low, ranging from 3.4 ± 0.5% in ‘Rubinstep’ to 1.3 ± 0.3% in ‘Elstar’. Cultivars ‘Red Aroma’ and ‘Asfari’ did not produce any fruit.

In 2023, the average fruit set, calculated from the mean values of various pollination combinations, was highest for the ‘Rubinstep’ (21%), followed by ‘Elstar’ (19.9%), ‘Red Aroma’ (18.3%), ‘Discovery’ (12.7%), and ‘Asfari’ (10.8%). Among pollination combinations tested with pollenizers, the ‘Rubinstep’ × ‘Katja’ combination achieved the highest fruit set that year, at 38.7 ± 5.5%. Among other mother cultivars, pollination combinations such as ‘Red Aroma’ × ‘Rubinstep’ (38.2 ± 3.2%), ‘Elstar’ × ‘Rubinstep’ (33.7 ± 2.6%), ‘Asfari’ × ‘Professor Sprenger’ (21.7 ± 2.8%), and ‘Discovery’ × ‘Red Aroma’ (20.3 ± 1.0%) also performed well. The lowest fruit set was observed in ‘Asfari’ × ‘Eden’ (9.0 ± 1.0%), followed by ‘Red Aroma’ × ‘Dolgo’ (11.3 ± 1.5%), ‘Discovery’ × ‘Summerred’ (15.0 ± 2.5%), ‘Elstar’ × ‘Red Aroma’ (15.3 ± 1.8%), and ‘Rubinstep’ × ‘Red Aroma’ (21.5 ± 1.2%). Open pollination produced much lower results, about 2 to 6 times lower than in 2022, depending on the pollen recipient cultivars. The pollen recipient ‘Red Aroma’ showed the highest fruit set at 23.2 ± 3.8%, while ‘Discovery’ had the lowest at 4.3 ± 0.5%. During self-pollination, fruit set was not observed in four maternal plants, except for ‘Rubinstep’, which had a rate of 1.5 ± 0.5%.

## 3. Discussion

### 3.1. In Vitro Pollen Germination and In Vivo Pollen Tube Growth

In previous studies, Florin [30] classified different apple and pear cultivars based on their pollen germination rates as poor (<30%), average (30–70%), or good (>70%). Additionally, pollen germinability in apple is much higher in diploid cultivars, most reaching at least 50% and very often exceeding 70%, while most triploid cultivars reach 10% viability or less [5]. In our study, the in vitro pollen germination test showed that pollen viability did not differ significantly between cultivars or years. The highest average pollen germination over two years was 87.8% for ‘Red Aroma’, while the lowest was 37.3% for ‘Summerred’. This range of in vitro pollen germination, from lowest to highest, has been reported by Javid and Rather [31], who found that the average pollen germination percentages across a set of apple varieties in India ranged from 56% to 95.5%. Similar to our study, De Albuquerque Junior et al. [32] observed pollen germination rates ranging from 59.6% to 73.2% across apple cultivars. In general, there is no standardized method for assessing pollen germination in vitro, particularly regarding thresholds, control cultivars, and criteria for determining pollen viability [8]. If the in vitro pollen germination rate exceeds 70% (averaged over two years), it is considered good. Therefore, ‘Red Aroma’, along with ‘Fryd’, ‘Discovery’, ‘Dolgo’, and ‘Rubinstep’, can be regarded as potentially successful pollenizers.

Several factors, including cultivar/genotype, flower age, time of collection, season, storage conditions, and the methodology used, influence in vitro pollen germination. Variation in pollen germination percentage and pollen tube length can be attributed to differences in pollen protein and carbohydrate content, which result from genetic variation among the studied cultivars [33]. Additionally, climatic conditions—particularly extreme temperatures, drought, and micronutrient or heavy metal imbalances—can substantially reduce pollen germination [34,35]. Moreover, interactions between external and internal factors during pollen development and tube growth can activate or inhibit enzyme systems, influencing pollen growth both in vitro and in vivo [36].

The progamic phase of fertilization comprises four stages: pollen germination, pollen tube growth into the stigma and ovary, and the release of male gametes into the egg apparatus [37]. In apple, pollen tube growth begins when pollen grains germinate on the funnel-shaped stigma surface, which captures pollen and facilitates hydration, germination, and pollen–stigma interactions [38]. All stigmas become receptive at the same time, regardless of the number of carpels [39]. The moist, papillate stigma surface and its secretions support germination and recognition, but receptivity declines as the stigma dries and turns brown. 

This study investigated the progamic phase of fertilization, focusing on pollen tube growth dynamics in the pistils of five apple cultivars after pollination with selected pollinizers, including self-pollination. Based on results from both years, cultivars ‘Discovery’, ‘Red Aroma’, and ‘Asfari’, as mothers, showed the highest pollen tube growth in 2022, meaning they had the highest percentage of pistils with penetration into the ovary locules across all tested pollination combinations (excluding self-pollination). By the sixth day, pollen tubes had penetrated 100% of pistils in ‘Discovery’ (with pollenizers ‘Dogo’, ‘Summerred’, ‘Katja’, and ‘Red Aroma’) in both years and in ’Red Aroma’ (with pollenizers ‘Dolgo’, ‘Discovery’, and ‘Katja’) in 2022. Additionally, individual pollenizers showed varying efficiencies across pollination combinations, with results that varied from year to year. For example, ‘Rubinstep’ crossed with ‘Red Aroma’ had 100% of pistils with pollen tubes that penetrated the ovary locules in 2022, whereas in 2023, that percentage was only 58.3% after 10 days of pollination. A similar pattern was observed in ‘Elstar’ pollinated with ‘Red Aroma’, with much smaller variation (83.3% in 2022 and 81% in 2023). Although pollen tube growth efficiency varies by year, these values still indicate that factors such as pollen quality, stigma receptivity, and tube activation and guidance can influence the process [40,41]. Based on the in vitro pollen quality test, it cannot be concluded that pollen germinability is directly linked to more efficient pollen tube growth in vivo.

Additionally, in vivo pollen tube growth is influenced by intercellular substances that nourish and guide the tubes through female tissues [42,43]. In addition to these factors, temperature and maternal genotype are well known to significantly affect pollen tube growth in vivo [9,44]. In our study, the temperature during pollen tube growth from the stigma to the carpelary locule was similar in both years (11.6 °C in 2022 and 11.8 °C in 2023), indicating that temperature is not the key factor for pollen tube growth in vivo. Conversely, research on the progamic phase in some apple cultivars that bloom later showed that each genotype responds differently to temperature changes, with these differences more noticeable during that period [45,46,47].

Regarding self-pollination, pollen tube penetration into the lower part of the style was confirmed in 9.1% of pistils in ‘Elstar’ in 2022. In 2023, in ‘Red Aroma’, the maximum pollen tube penetration into the lower part of the style was observed in 70% of pistils. Most pollen tubes exhibited looping, curling, and thickening, indicating self-incompatibility. Therefore, all cultivars are considered self-incompatible.

### 3.2. Development and Viability of Embryo Sac

Successful fruit set in apple depends on embryo sac development and longevity, the efficiency of the progamic phase, and fertilization success [48]. Apple ovules contain monosporic, eight-nucleate *Polygonum*-type embryo sacs [49]. The embryo sac regulates pollen tube guidance, gamete fusion, and embryo formation [50]. Synergids facilitate male gamete movement [51], whereas antipodes are short-lived and degenerate before fertilization [52]. In this study, one synergid usually degenerated 3 days after BFF in ‘Rubinstep’ and ‘Elstar’, and 6 days after BFF in ‘Discovery’, ‘Red Aroma’, and ‘Asfari’ (at the 4-nucleate stage of the embryo sac). Polar nuclei may partially fuse, forming a central nucleus, before fertilization [53].

The functional female gametophyte, whose development is genotype dependent, plays a crucial role in fertilization and fruit set [54,55]. Within the embryo sac, the egg cell and polar nuclei participate in double fertilization. On the tenth day after BFF, cultivars ‘Discovery’, ‘Rubinstep’, and ‘Elstar’ showed higher percentages of functional embryo sac stages in 2023 than in 2022, whereas ‘Red Aroma’ and ‘Asfari’ showed the opposite trend (Figure 8). Across all cultivars and both years, embryo sac viability declined sharply between the sixth and tenth days after BFF. The 8-, 5-, and 4-nucleate stages (cumulative sum) followed a parabolic viability trend. Strong correlations (R^2^ = 0.94–0.99) confirmed close relationships between embryo sac stages and days after flowering. Similar temporal patterns were reported in pear [26] and plum cultivars [27]. In stone fruits, viable embryo sacs may disappear by the sixth day after BFF, persist until day twelve, or develop too slowly, leaving many ovules without embryo sacs [56,57]. In our study, a higher initial number of functional stages at full bloom did not ensure greater embryo sac viability ten days after BFF. These findings indicate that full-bloom megagametophyte development does not guarantee fruit set, as abundant functional ovules may still result in low yields. Hence, high productivity requires more viable ovules than pollen grains.

### 3.3. Fertilization Success and Fruit Set

Fertilization success mainly depends on events during the differentiation and maturation of male and female gametes, the progamic phase, syngamy, embryogenesis, and post-fertilization processes, which are influenced by both internal and external factors, primarily ecological conditions, such as environmental adaptation. It means that male and female gametes must be in the same phase of the cell cycle to achieve successful mating [58]. In this study, the appearance of a fertilized egg cell and early embryogenesis in the embryo sac can be observed as early as the tenth day after BFF, depending on the cultivar and year. On later dates following the BFF, all cultivars in both years showed a higher percentage of embryo sacs with fertilized egg cells and early embryogenesis in 2022 than in 2023. Studies on pear and plum under similar climatic conditions have shown that fertilization success is closely related to average temperatures, which influence male and female gametes, particularly during the progamic phase and syngamy [26,27]. In our study, the mean daily temperature during the 21 days following full bloom was similar in both years (12.7 °C in 2022 and 13.6 °C in 2023), indicating that temperature did not contribute to the observed differences in fertilization success between years. It is assumed that the key factors for fertilization success (embryo sac viability and the in vivo growth rate of pollen tubes) reflect the genotypic specificity of cultivar-pollenizers and cultivar-pollen recipients with respect to specific temperatures [59].

Pollination is a decisive factor influencing fruit set, growth, fruit quality, and seed development in temperate fruit species, particularly in Malus domestica Borkh. cultivars. The success of fertilization, reflected in the transition of the ovary into a developing fruit, depends mainly on the efficiency of the progamic phase, including pollen transfer, pollen tube growth through the style, and ovule fertilization [41]. The outcome of these processes is highly sensitive to environmental conditions and biological interactions. Weather fluctuations, pollinator activity, inter-cultivar compatibility, and flowering synchrony can markedly affect fertilization efficiency, nutrient distribution, and the incidence of early flower abscission [47,60]. Periods of water stress during the summer months (June–August) may further contribute to fruit drop; however, under the climatic conditions of Western Norway, variation in fruit set between years appears more strongly associated with temperature and pollination dynamics than with drought stress. Similar interannual variation has been reported in pear and plum cultivars under comparable conditions [22,23]. Furthermore, biennial bearing remains a major constraint in apple production, significantly affecting fruit-set patterns from year to year. In biennial-bearing cultivars, more than 90% of terminal buds may produce fruit in the ‘on’ year, whereas in annual-bearing cultivars, the proportion rarely exceeds 20% [61,62].

The average fruit set across all pollination variants and pollenizers for the tested pollen-recipient cultivars was higher in 2022 (23.2%) than in 2023 (16.3%). This trend was consistent in ‘Discovery’, ‘Rubinstep’, ‘Red Aroma’, and ‘Asfari’, while only ‘Elstar’ showed the opposite pattern. Excluding in vitro pollen-germination values, in vivo pollen-tube growth through the pistil showed more favorable dynamics in 2022 across most recipient cultivars and pollination combinations. This was particularly evident on the sixth day after BFF but remained detectable ten days after BFF. Higher fertilization success rates, meaning embryo sacs with fertilized eggs and early embryogenesis in 2022 compared to 2023, may influence these yearly differences in fruit set. This is especially noticeable with ‘Elstar’, ‘Red Aroma’, and ‘Asfari’, which had fertilization success rates of 100% of embryo sacs 21 days after BFF in 2022, compared with 80%, 71.4%, and 62.5%, respectively, in 2023. In these cultivars, the average fruit set for ‘Red Aroma’ was 33.4% in 2022 and 18.3% in 2023, while for ‘Asfari’ it was 26.1% in 2022 and 10.8% in 2023. Unlike these cultivars, ‘Elstar’ achieved a 100% fertilization success rate in 2022, with a higher average fruit set in 2023 (19.9%) than in 2022 (14.3%). These findings suggest that reproductive processes in 2022 were more efficient in some cultivars, meaning that improved dynamics of pollen tubes during the progamic phase and higher fertilization success contributed to higher fruit set, even though environmental conditions during flowering were similar in both years. On the other hand, in some cultivars (‘Elstar’), these parameters did not produce this synergistic effect on fruit set. On the other hand, aside from depending on temperature, the success of the pollenizer can also depend on the influence of the pollen recipient cultivar, which can affect fruit set, as observed in other fruit species [23,63]. With some other apple varieties, June drop caused by water deficit can significantly reduce fruit set [28]. Otherwise, the June drop is related to protein synthesis, mineral nutrition, the number of fertilized ovules per fruit, and embryo abortion [64]. Data collected over two years show that the average fruit set, calculated across all open-pollination combinations of recipient cultivars, varies, with larger differences observed in 2022 (25.2%) than in 2023 (11.2%). This suggests that, unlike hand pollination, the examined cultivars have different levels of pollen receptivity during full flowering and do not receive the same amounts and qualities of pollen, which affects their fruit set over the years.

When considering the best pollenizers for each cultivar, ‘Discovery‘ had the highest average two-year fruit set with ‘Red Aroma’ (21%) and ‘Dolgo’ (20.9%). For ‘Rubinstep’, the most effective pollenizers in 2022 were ‘Red Aroma’ (38.7%) and ‘Discovery’ (22.3%), while in 2023 the highest fruit set was achieved with ‘Katja’ (38.7%) and again ‘Discovery’ (30.1%). For ‘Red Aroma’, the top pollenizers in 2022 were ‘Discovery’ (58.2%), ‘Katja’ (46.5%), and ‘Rubinstep’ (46.0%). In 2023, ‘Red Aroma’ again had the highest values, followed by ‘Rubinstep’ (38.2%) and ‘Discovery’ (20.3%). For ‘Elstar’, ‘Rubinstep’ (22.5%) and ‘Red Aroma’ (15.2%) were the most effective pollenizers in 2022, whereas in 2023, the highest fruit set again came from ‘Rubinstep’ (33.7%), followed by ‘Asfari’ (32%). For ‘Asfari’, the most effective pollenizers in 2022 were ‘Eden’ (42.9%) and ‘Rubinstep’ (38.7%), while in 2023 the highest fruit set was with ‘Professor Sprenger’ (21.7%) and ‘Rubinstep’ (16.8%). Our results are consistent with those reported by Jahed and Hrist [12], who noted that, in some apple cultivars, fruit set can vary widely with pollenizers and years (6.7%—86.7%). Apple trees generally produce more flowers than are required; only 5–10% of fertilized flowers are needed for a good yield. However, abundant flowering doesn’t guarantee high productivity, as many flowers or fruits may drop from flowering until four weeks later [65].

In our study, all examined factors—pollination variant/pollenizer, year of study, and their interactions —had a significant effect on fruit set. These variations highlight the complex interactions among environmental conditions, cultivar physiology, and reproductive biology in determining fruit-set stability. This is evident in aspects of reproductive biology, including pollen germination, embryo sac longevity, the progamic phase, and fertilization success, all of which relate to fruit set. In some cases, and with some cultivars, high values of these parameters do not always result in high fruit set. The apple genotype influences bud development into fruit, thereby affecting overall yield potential; plant hormones regulate fruit set, growth, and development. Their balance affects flowering, fruit size, and the total number of fruits [66].

During self-pollination, fruit set was observed in 2022 for ‘Discovery’ (2.4%), ‘Rubinstep’ (3.4%), and ‘Elstar’ (1.3%), but only in ‘Rubinstep’ (1.5%) in 2023. Similar values were obtained with ‘Fuji’ and ‘Golden Delicious’, which produced only 1% and 1.8% fruit set, respectively, after self-pollination, while ‘Elstar’ (7%) and ‘Idared’ (12.3%) sometimes showed higher values [67]. Our data on the dynamics of pollen tube growth in vivo and fruit set after self-pollination indicate that the tested cultivars are practically self-incompatible. Most apple cultivars are primarily self-incompatible, with few capable of self-pollination. 

### 3.4. Overlapping in Flowering Time

Flowering synchrony between pollen recipients and pollenizers is crucial for successful pollination, fertilization, and fruit set. Because most apple cultivars are self-incompatible, compatible pollenizers are essential for effective cross-pollination and improved yield. Cultivars with overlapping flowering periods serve as efficient pollen donors, as greater synchrony enhances siring success [68].

Considering only the two most effective pollenizers, as determined by the highest fruit set percentages and the duration of flowering overlap, interannual variation was observed. In 2022, the ‘Discovery’ × ‘Dolgo’, ‘Elstar’ × ‘Rubinstep’, and ‘Asfari’ × ‘Dolgo’ combinations had the shortest overlap of eight days, while ‘Rubinstep’ × ‘Discovery’ had the longest at twelve days. In 2023, the shortest overlap (eight days) occurred among ‘Discovery’ × ‘Red Aroma’, ‘Rubinstep’ × ‘Discovery’, and ‘Red Aroma’ × ‘Discovery’, whereas the ‘Red Aroma’ × ‘Rubinstep’ combination had the longest overlap (thirteen days). In practice, cultivar combinations with a longer overlapping flowering period should be preferred as pollenizers.

## 4. Materials and Methods

### 4.1. Plant Material and Experimental Design

The five apple cultivars ‘Discovery’, ‘Rubinstep’, ‘Red Aroma’, ‘Elstar’, and ‘Asfari’, all diploid (2n), were used as pollen recipients, in addition to their self- and open pollination, and for embryological study in both unpollinated and open pollinated variants. The cultivars mentioned above, except for ‘Elstar’, along with the cultivars ‘Dolgo’ (crab apple), ‘Summerred’, ‘Katja’, ‘Professor Sprenger’ (crab apple), ‘Fryd’, and ‘Eden’, served as pollenizers in the following pollination combination scheme:
‘Discovery’ × ’Dolgo’, ‘Discovery’ × ‘Summerred’, ‘Discovery’ × ‘Katja’, ‘Discovery’ × ‘Red Aroma’;‘Rubinstep’ × ‘Dolgo’, ‘Rubinstep’ × ‘Discovery’, ‘Rubinstep’ × ‘Katja’, ‘Rubinstep’ × ‘Red Aroma’;‘Red Aroma’ × ‘Dolgo’, ‘Red Aroma’ × ‘Discovery’, ‘Red Aroma’ × ‘Katja’, ‘Red Aroma’ × ‘Rubinstep’;‘Elstar’ × ‘Rubinstep’, ‘Elstar’ × ‘Red Aroma’, ‘Elstar’ × ‘Asfari’, ‘Elstar’ × ‘Professor Sprenger’;‘Asfari’ × ‘Rubinstep’, ‘Asfari’ × ‘Eden’, ‘Asfari’ × ‘Fryd’, ‘Asfari’ × ‘Professor Sprenger’;


Each cultivar was represented by at least 20 trees, all planted in an experimental apple orchard in Lofthus, Western Norway, at latitude 60°19′12.5″ N and longitude 6°39′13.4″ E, 12 m above sea level. The orchard was established in 2019 using two-year-old feathered trees. All cultivars were grafted onto M9 rootstock. Trees were spaced 3.5 × 1 m apart and trained as slender spindle trees. Selected trees for the experiment consistently bloomed, reflecting the orchard’s average bloom intensity and tree size. No thinning was performed. Orchard management included maintaining grass between the rows and a 1-m-wide strip of vegetation-free space within the rows. During the season, the trees were fertigated daily from drip lines along the rows, based on evaporation rates. The research took place over two consecutive years (2022–2023).

### 4.2. Climate Conditions and Flowering Time

Western Norway has mild winters and cool summers. Weather systems developing over the Atlantic Ocean often bring clouds, rain, and wind throughout the year. Although the Gulf Stream helps moderate temperatures, spring cold snaps still occur. In the Hardanger region, most precipitation falls in winter, while summers are typically relatively dry, necessitating irrigation for fruit trees. The yearly average temperature is 7.6 °C, and total precipitation averages 1705 mm (average data 1991–2021). During the growing season (May–September), the average temperature is 13 °C, and rainfall is 459 mm. Over two years, daily mean, maximum, and minimum temperatures, along with precipitation in millimeters, were recorded during the flowering period of the tested apple cultivars. 

The flowering phenophase for each tested cultivar was recorded using the BBCH scale, which classifies plant phenological growth stages [69]. The onset of flowering (BBCH stage 61) was recorded when approximately 10% of the flowers had opened. Full bloom (BBCH stage 65) was recorded when 50% of the flowers were open, and the end of flowering was recorded when most petals had fallen (BBCH stage 67).

### 4.3. Pollen Germination In Vitro

Flowers were collected during the late balloon stage from all sides of the trees of the studied apple cultivars used as pollenizers. Anthers were sampled under laboratory conditions and stored in paper boxes at 20 °C until they opened to release pollen grains (24–48 h). Pollen from all genotypes was cultured in three Petri dishes using a medium composed of 1% agar and 14% sucrose. After incubation for 24 h at 20 °C, the number of germinated pollen grains was counted under a Leica DM LS light microscope (Leica Microsystems Wetzlar GmbH, Wetzlar, Germany) at 100× magnification. The number of germinated grains was determined in three microscopic fields of view (each covering 100 pollen grains) per Petri dish. The germination percentage was calculated as the average across the nine microscopic fields. Grains were considered germinated if their tube was longer than their diameter [70].

### 4.4. Pollination Procedure

During the balloon stage, branches with flowers from 2–3 trees or pollination combinations of pollen recipient cultivars such as ‘Discovery’, ‘Rubinstep’, ‘Red Aroma’, ‘Elstar’, and ‘Asfari’ were selected and marked. Open flowers were removed, and about 250–300 were emasculated and marked for each cross combination. The exact number of flowers was designated for self-pollination and open pollination. For artificial (hand) pollination, ‘balloons’ from cultivar donors were collected, and the anthers were removed in Petri dishes, left open at room temperature to dry and release pollen grains. Once the pollen was shed, the dishes were closed and stored at 4 °C. Pollination was carried out 24 to 72 h after emasculation, during the early hours when the mother trees were in full bloom (with nearby branches bearing fully open flowers, when anthers start changing color, and when stigma secretion was visible). Hand pollination of emasculated flowers involved dipping a finger into the pollen-filled Petri dish and touching the exposed stigma twice. Pollination was performed on flowers that were fully open on two- and three-year-old branches. For this study, the following pollinations were conducted: self-pollination of ‘Discovery’, ‘Rubinstep’, ‘Red Aroma’, ‘Elstar’, and ‘Asfari’, as well as pollination with different pollenizers according to the previously mentioned pollination-combination scheme. 

### 4.5. Pollen Tube Growth in the Pistil

Thirty pistils from each pollination combination (three repetitions × 10 pistils) were collected on the third, sixth, and tenth days after the beginning of full flowering (BFF) and placed in FPA solution (90:5:5 ratio of 95% ethanol, propionic acid, and formalin (40% formaldehyde). Fixed materials were stored at 4 °C until processing and stained with aniline blue to visualize pollen tube growth, following the methods of Preil [71] and Khö and Baer [72]. For short-term squash preparations, the style (including five separate styles and the joint style) was separated from the ovary and covered with a coverslip. The ovary was then sliced with a razor blade to observe pollen tube penetration into the ovary locules. The dynamics of pollen tube growth through the upper, middle, and lower parts of the styles into the ovary locules are presented as the percentage of pistils in which pollen tubes reached these parts at their maximum, for each pollination combination and fixation day. Microscopes Leica DM LS in fluorescence mode with filters A (wavelength 340–380 nm) and I3 (wavelength 450–490 nm), equipped with a Leica Flexcam C3 (Leica Microsystems, Nussloch, Germany) color camera, were used to observe and capture images of pollen tube growth in vivo (magnification 50×).

### 4.6. Histology Preparations for Embryology

Two groups of flowers were prepared to study the embryology of the cultivars ‘Discovery’, ‘Rubinstep’, ‘Red Aroma’, ‘Elstar’, and ‘Asfari’. Approximately 250 to 300 flowers per cultivar were emasculated and left unpollinated to assess embryo sac functionality (development and viability). Emasculated flowers were bagged to prevent accidental pollination. Additionally, a second group of 250–300 flowers per cultivar was counted and tagged, left for open pollination to study fertilization success. Flowers from both groups (three replicates × 5 pistils) were collected at the beginning of full flowering (BFF) and on the 3rd, 6th, 10th, 14th, and 21st days after BFF. Ovaries were fixed in FPA and stored at 4 °C. The tissue was then dehydrated through a series of ethanol washes and embedded in paraffin blocks, which were sectioned longitudinally at 10 µm using a Leica RM 2155 rotary microtome (Leica Microsystems, Nussloch, Germany). Sections mounted on glass slides were stained with safranin, crystal violet, and light green SF, as described by Gerlach [73]. Permanently stained sections were observed and photographed using an Optic 900T light microscope with a TCA1000-C color camera (Colo LabExperts, Polje ob Sotli, Slovenia) at 100×, 200×, and 400× magnification. 

### 4.7. Final Fruit Set

A total of 250–300 flowers from each pollination combination (including open and self-pollination) were selected to assess fruit set. The final fruit set was recorded just before harvest (BBCH 79) and fruit-set percentages were calculated as the number of fruits formed or harvested per 100 flowers.

### 4.8. Statistical Analysis

The data (pollen germination, pollen tube length in vitro, and fruit set) were analyzed using Fisher’s analysis of variance (ANOVA) with a two-factor design, and the F-test was applied at the significance level *p* ≤ 0.05. When the F-test was significant, differences between means were assessed using the Tukey test, also at *p* ≤ 0.05. Statistical analyses were conducted using SPSS for Windows, Version 8.0 (SPSS Inc., Chicago, IL, USA).

Regression analysis identified an optimal relationship between the functional stages of embryo sacs and the number of days after flowering begins. The Stats.Blue online statistical software was used to perform regression analyses and calculate the correlation coefficient (R^2^).

## 5. Conclusions

Norwegian apple production faces challenges such as climate issues, flowering asynchrony, and a limited number of suitable cultivars and pollenizers. This study examined flowering time, pollen germination, pollen tube growth, embryo sac development, fertilization, and fruit set in five cultivars (‘Discovery’, ‘Rubinstep’, ‘Red Aroma’, ‘Elstar’, and ‘Asfari’) across different pollination combinations, including open and self-pollination during the 2022–2023 seasons. Studying pollen tube growth, embryo sac viability, and early embryological stages can maximize yield and fruit quality. Results showed that in vitro pollen viability does not always correspond to in vivo pollen tube growth, which was more dependent on cultivar than temperature. Regarding embryo sac viability, the trend in functional stages (8-, 5-, and 4-nucleate) varied by cultivar and study year. Viability declined from day six to ten after the beginning of full flowering, with no functional stages afterward. Based on the results obtained, the recommended pollenizers are as follows: for ‘Discovery’—‘Red Aroma’ and ‘Dolgo’; for ‘Rubinstep’ ‘Red Aroma’, ‘Katja’, and ‘Discovery’; for ‘Red Aroma’—‘Rubinstep’, ‘Discovery’, and ‘Katja’; for ‘Elstar’—‘Rubinstep’, ‘Asfari’, and ‘Red Aroma’; and for ‘Asfari’—‘Eden’, ‘Professor Sprenger’, and ‘Rubinstep’. Considering the longest average flowering overlap among the pollenizers listed above, the best pollenizer for the each of the studied cultivars was as follows: for ‘Discovery’—‘Red Aroma’; for ‘Rubinstep’—‘Red Aroma’; for ‘Red Aroma’—‘Rubinstep’; for ‘Elstar’—‘Red Aroma’; and for ‘Asfari—‘Professor Sprenger’. These findings are crucial because synchronized flowering between the main cultivar and pollenizers within the orchard can ensure high, stable, and economically viable yields in the specific climate of Western Norway.

## Data Availability

The original contributions presented in the study are included in the article. Further inquiries can be directed to the corresponding author.
